# Universal representations of evaporation modes in sessile droplets

**DOI:** 10.1371/journal.pone.0184997

**Published:** 2017-09-15

**Authors:** Angkur Jyoti Dipanka Shaikeea, Saptarshi Basu, Abhishek Tyagi, Saksham Sharma, Rishabh Hans, Lalit Bansal

**Affiliations:** 1 Department of Engineering Science, University of Oxford, Oxford, United Kingdom; 2 Department of Mechanical Engineering, Indian Institute of Science, Bangalore, India; North China Electric Power University, CHINA

## Abstract

In this work, we provide a simple method to represent the contact line dynamics of an evaporating sessile droplet. As a droplet evaporates, two distinct contact line dynamics are observed. They are collectively known as modes of evaporation, namely Constant Contact Radius (CCR) and Constant Contact Angle (CCA). Another intermediate mode—Stick-Slide (SS) or mixed mode is also commonly observed. In this article, we are able to provide a graphical representation to these modes (named as MOE plot), which is visually more comprehensive especially for comparative studies. In addition, the method facilitates quantitative estimation for mode of evaporation (named as MOE fraction or MOE_f_), which doesn’t exist in literature. Thus, various substrates can now be compared based on mode of evaporation (or contact line dynamics), which are governed by fluid property and surface characteristics.

## Introduction

Droplet evaporation is ubiquitous to applications ranging from biophysics to engineering. Miniaturization involving droplet architecture is common for lab-on-chip studies [1–3] and applications like droplet-based microfluidics [4–8], micro-scale heat transfer [9–11], surface patterning [12–15] to name a few. Thereby droplet studies have gained significant momentum in the present decade. One of the interesting areas is the dynamics of the three-phase contact line (CL) at the ambient-fluid-substrate interface. When a drop is deployed on a surface (substrate), it takes spherical cap geometry (due to minimum surface energy) and the contact angle (CA) formed at the interface is determined by Young’s law. As evaporation proceeds, the drop diminishes in volume. Unless otherwise disturbed by external factors like droplets in a colony [16], evaporating droplets are generally symmetrical and should maintain the initial equilibrium CA. However, due to surface roughness and particle deposition (particle laden droplets) at the CL, most of the drops pin to the substrate. With evaporation, the pinned CL is stressed and after some reduction in CA (contact angle hysteresis, CAH), the CL begins to slip. The former is known as CCR mode while the latter is known as Stick-Slide (SS) or mixed mode. This consequently affects the deposition pattern (like coffee ring effect on hydrophilic substrates [17–18] and buckled shells on hydrophobic substrates [19–20]). Furthermore, a droplet in CCR mode evaporates faster than SS, followed by CCA [21]. Hence, mode of evaporation is of significant importance in not only determining the evaporation lifetime but also particle deposition patterns.

**Fig 1:**
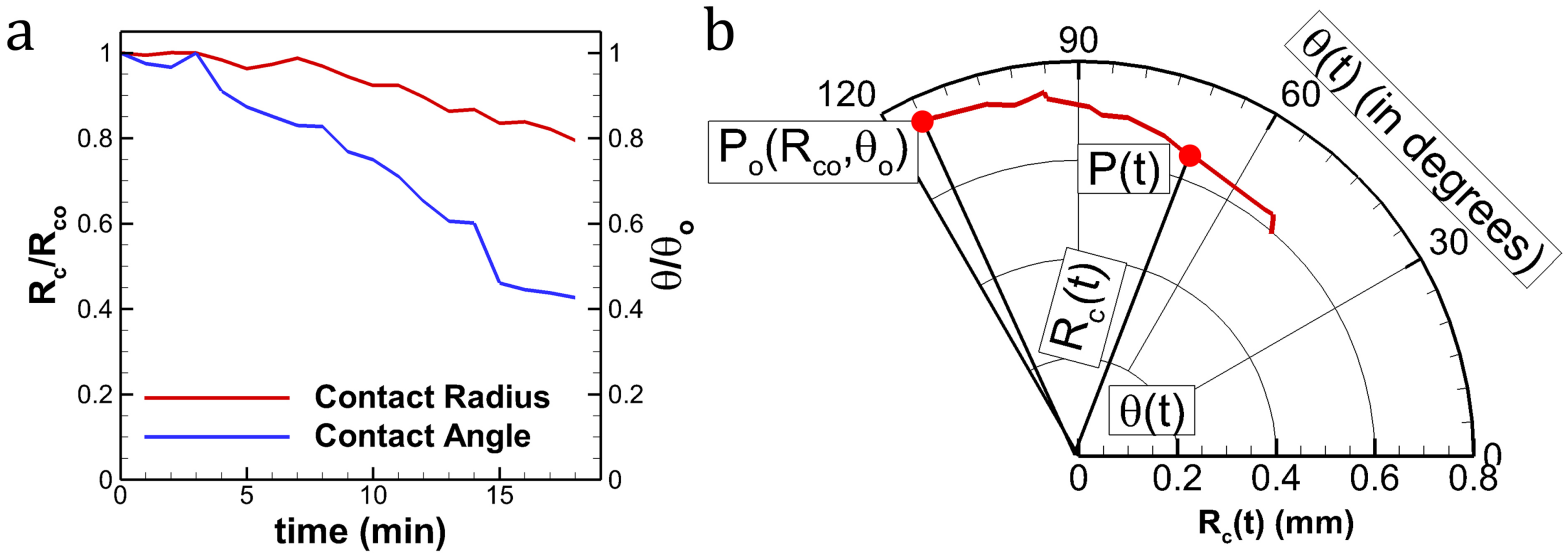
Droplet contact line dynamics. (a) Normalized CR, CA vs. time. (b) Equivalent polar plot (MOE plot) of (a) with nomenclature.

**Fig 2:**
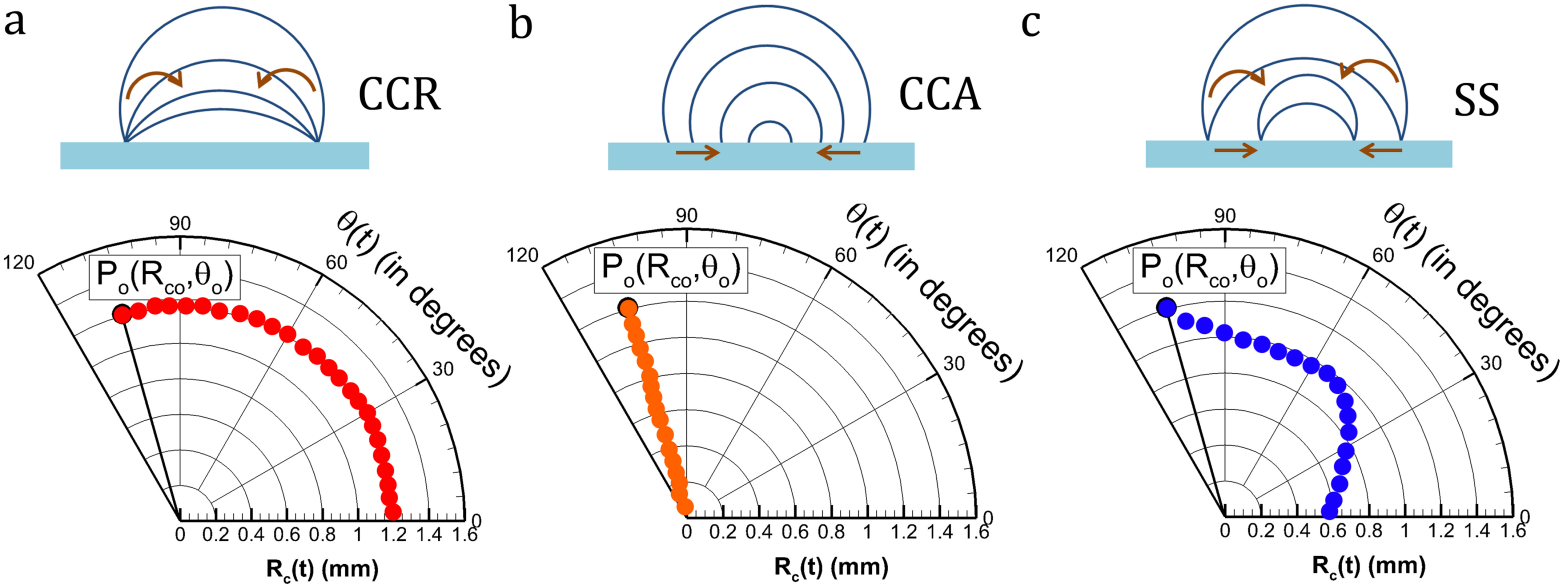
Theoretical sketch for MOE plots in three different modes. (a) CCR (b) CCA (c) SS.

In present literature, contact radius (CR) and CA are usually plotted on a rectangular coordinate system. Although the data is represented completely and CCR, CCA or SS can be identified, the graphical interpretation is clumsy (Fig 1a). To simply this, a single trajectory formed by the temporal coordinate P(R_c_(t), θ(t)) of the evolving droplet is plotted on a polar coordinate system. Here, P is the coordinate of the instantaneous contact radius and contact angle. Such a plot is named as MOE plot (Fig 1b). Here, R_c_(t) is the CR and θ(t) is the CA at any instant ‘t’ (t>0). By following this convention, CCR is a circular arc (Fig 2a) and CCA is an arrested angle (Fig 2b) on the polar plot. SS shows both angular and radial variations (Fig 2c). In addition to the visual convenience; when multiple data are plotted on the same figure, comparison across substrates based on mode of evaporation becomes clear. This was not possible in the existing plot style as available in the literature. Furthermore, we define a parameter called mode of evaporation fraction or MOE_f_ to estimate the tendency of a droplet to be in a particular mode which will be discussed in the following sections.

## Methods

Experiments on droplet evaporation are performed on a horizontal platform surrounded by high speed IDT camera (fitted with Navitar lens), thermometer, hygrometer, diffuser and cold light source. Experiments are conducted with deionized water droplets deployed on three different substrates with varying degrees of CL pinning i.e. modes of evaporation. The substrates—polydimethylsiloxane (PDMS), Gas Diffusion Layer (GDL), superhydrophobic (SH) are prepared by standard protocol as explained in SI (Substrate Preparation). Although experiments are conducted for different initial volumes ranging from 0.5μl to 3μl, data reported here corresponds to 3μl droplet unless otherwise specified. Ambient conditions of experiments are maintained at 25°C and 45% RH. The setup is kept isolated from external disturbances. Once experiments are done, the images are analyzed using imageJ software to calculate contact radius and contact angle. This is done by delineating the interface of droplet with a sphere, adhering to the spherical cap approximation (for droplet contact radius less than capillary length scale; *l*_*c*_ = (ρgh/σ)^1/2^ ~ 2.7 mm [22]). The contact angle calculated in this way is nearly constant from all views, due to homogeneity of surface. In case of heterogeneous or hierarchically structured surfaces [23], average contact angle along the contact line could be taken to reproduce this MOE plot. Finally, the data analysis on area under MOE plots is performed using Python/MATLAB codes.

## Results and discussion

**Fig 3:**
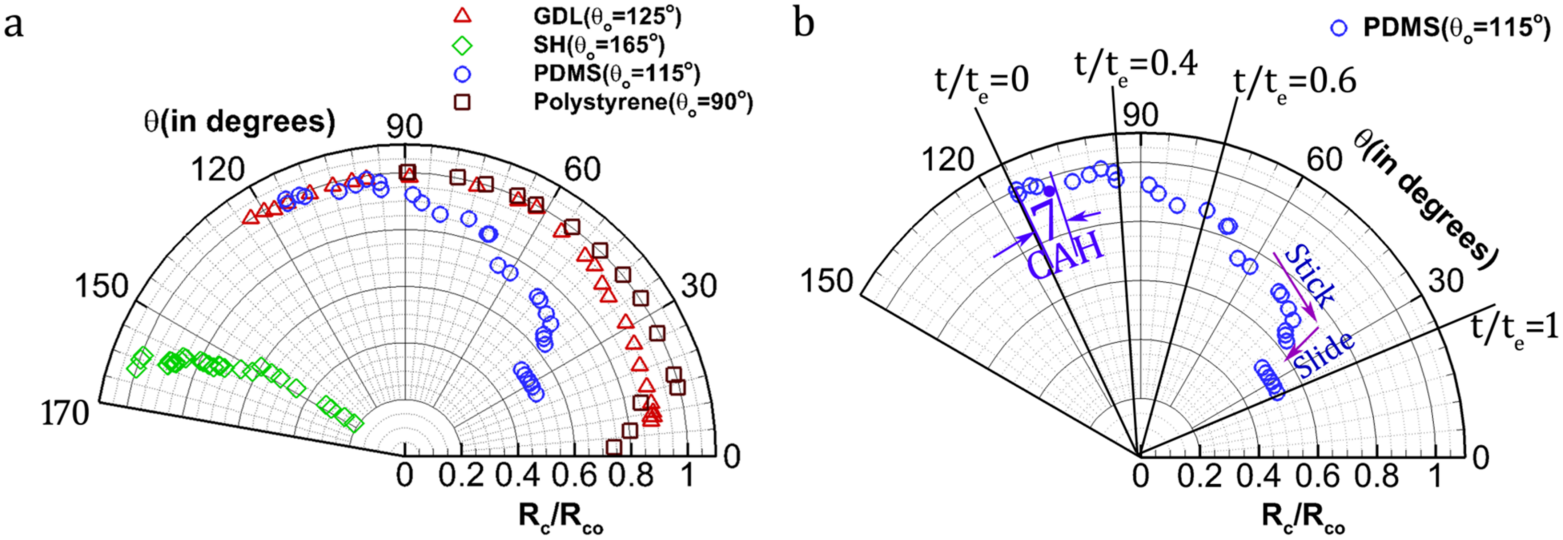
MOE plots. (a) For different substrates (hydrophobic and hydrophilic) with three distinct modes of evaporation. (b) For PDMS only. Non-dimensional time coordinates (t/t_e_) are also marked, where t_e_ is evaporation duration. Standard deviation error across repeated trials is within 4%.

Fig 1a shows the temporal variation of CA (θ(t)) and CR (R_c_(t)) in a conventional rectangular coordinate system vis. a vis. with time (t) on the x-axis and dependent variables R_c_(t) and θ(t) on the y-axes. Here, the variables are non-dimensionalised by their respective maximum values. This is for clarity in understanding and uniformity in visual demonstration (dependent variables on a scale 0 to 1). The approach adopted in this article uses a polar coordinate system with θ(t) on the angular direction and R_c_(t) on the radial axis in the same plot (Fig 1b). For a strictly CCR or CCA mode of evaporation, the hypothetical plots are provided in Fig 2a and 2b with insets showing the respective geometrical variation of the droplet shape during its lifetime of evaporation. For a more realistic droplet, the CL dynamics is mostly a combination of both, and /or temporally switches between CCR and CCA (Fig 2c). This concept of representing CR and CA on a polar coordinate system provides simplicity in comparison (qualitative as well as quantitative) based on the CL dynamics. To understand this cohesively, Fig 3a is provided with two extreme modes of CL dynamics taken from experiments, i.e. CCR and CCA (in ~90% of the droplet lifetime). Another drop following the intermediate mode, i.e. SS is also plotted on the same figure. The MOE plot for PDMS is shown separately (Fig 3b) to elucidate a few more advantages. The extent of variation in the respective dynamic variable (CA in CCR and CR in CCA) within each of these modes can be observed. For instance, for the initial CCR mode, CA decreases by ~20° which is beyond its CAH (~7°) value. Thereafter, both CA and CR vary simultaneously (SS mode). Towards the end, the CL undergoes stick-slip motion with short residence in CCR and CCA modes. Thus, departure from CL pinning is not strictly determined by CAH. Thereby, modes of evaporation vary temporally during the droplet lifetime. In addition, the time stamps at some of the inflexion points (CCR ↔ CCA) are also marked. It shows that CCR has a relatively faster evaporation rate as was theoretically proposed by Stauber et. al. [21]. Such information was graphically inconvenient to conclude from a rectangular coordinate plot. The CR values in these plots are non-dimensionalised by the initial CR (R_c_(t = 0) = R_co_) for the simplicity of beginning the plots at a constant radial value (equal to 1).

**Fig 4:**
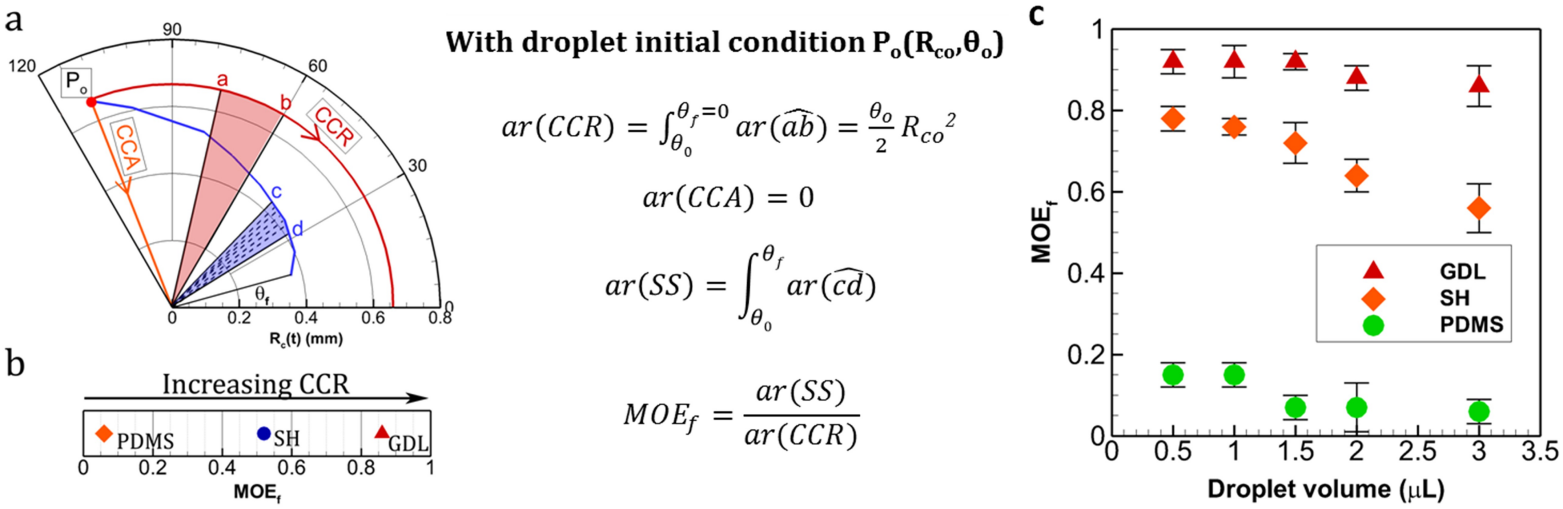
Mode of evaporation factor (MOE_f_). (a) Determination of MOE_f_ using area measurements from MOE plot. Here, $\text{ar}(\hat{\text{xy}})$, stands for area of the sector x0y. As $\hat{\text{cd}}$ is a non-circular arc, it is segmented into small sectors at the data points between c and d. Circular arc assumption is applied to each sector and area of sector c0d is obtained as sum of all. See S1 Supporting Information for details about area calculation ar(SS) (b) MOE_f_ for the substrates in Fig 3. (c) MOE_f_ plot for droplets with different initial volumes.

**Fig 5:**
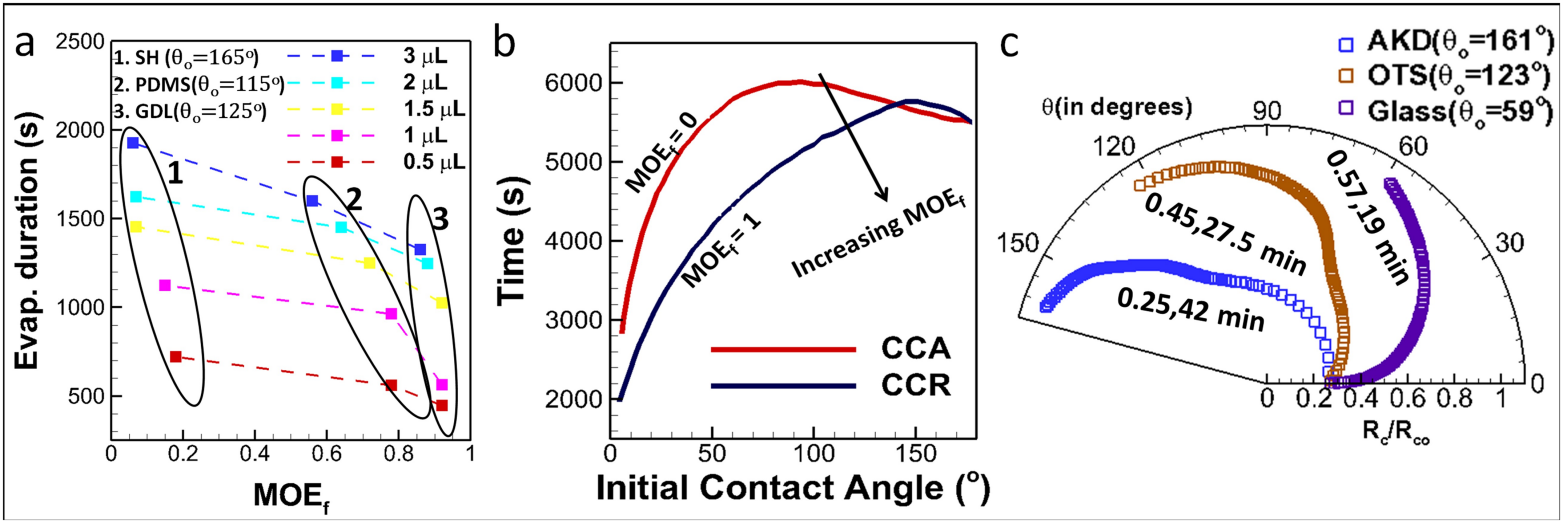
Physical significance of MOE_f_. (a) Variations in evaporation duration for different MOE_f_ values (experiments from three different substrates with different initial volumes) (b) Theoretical plot for variations in droplet evaporation time for strictly CCR and CCA modes in all possible initial contact angles (reprinted by permission from [24]). (c) MOE plot and corresponding MOE_f_ with evaporation time for data obtained from Shin, Dong Hwan, et al. [25]).

MOE plots can be helpful in quantifying modes of evaporation. This is especially significant to compare droplets across different substrates when undergoing SS. Firstly, the area of the sector enclosed by the trace of the plot between the two axes (θ = 0 and θ = θ_f_) is obtained as follows (Fig 4a). For CCR, the plot is a circular arc with radius equal to CR during CCR. Hence, area is equal to the area of the sector, given as (Δθ/2). R_c_^2^ (where, Δθ is the angle swept during CCR and R_c_ is the constant CR). For CCA, this area is zero. For SS, the plot follows a non-circular arc and hence it is split into small approximated circular-arc sectors (connected by data points) and then added. The higher is the number of data points, the more accurate is the area approximation. It is clear that area is maximum for CCR (ar(CCR)) and zero for CCA (ar(CCA) = 0) for any given initial conditions. For SS, the value of this area (ar(SS)) lies between CCR and CCA. Next, the area value is divided by the corresponding CCR area (ar(CCR)) and obtained as a fraction. This fraction is named as MOE fraction or MOE_f_, which gives the relative residence in CCR or CCA during the lifetime of droplet evaporation. For instance, if MOE_f_ is 0.86, it suggests that during droplet evaporation, the CL has nearly 86% tendency of pinning (CCR) and 14% (= 1-MOE_f_) towards CCA. MOE_f_ values 1 and 0 correspond to CCR and CCA respectively. MOE_f_ values for the three given substrates are plotted in Fig 4b. See S1 Supporting Information for steps in calculating ar(SS). CL pinning increases with decreasing droplet volume as inferred from MOE_f_ values shown in Fig 4c. A higher MOE_f_ signifies greater residence in CCR than CCA. Furthermore, from the previous works reported by Stauber et al. [21] and Picknett et al. [24], it is clear that evaporation duration decreases with increase in CCR (for initial contact angle < 145°) (as shown in Fig 5). This is due to the fact, in CCA mode there is continuous decrease in evaporation flux till it becomes zero however, in CCR mode evaporation flux achieves a constant value thereby resulting in faster evaporation [24]. Thus, a droplet-substrate combination with higher MOE_f_ would evaporate relatively faster (for the same initial volume of droplet, and experimental conditions in temperature and humidity). This is in corroboration with our experimental data shown in Fig 5a.

Mode of evaporation fraction (ratio of area under R vs. θ plots in Polar coordinates; $=\frac{experimental}{theoretical\;CCR}$) is related to three phase contact line pinning or slipping. A greater MOE_f_ signifies greater tendency towards contact line pinning and vice versa. For example, if we have MOE_f_ = 1, it indicates that the droplet is pinned for entire duration of its lifetime while MOE_f_ = 0.8 denotes reduction in pinning duration (CCR mode) by 20%. This provides a universal way of representing/quantifying the degree of pinning irrespective of initial volume and initial equilibrium contact angles (substrates of different hydrophobicity). This in turn facilitates the understanding about effect of contact line pinning on total evaporation duration which was qualitatively found as t_CCR_<t_SS_<t_CCA_ for initial contact angle < 145° where t_CCR_, t_SS_ and t_CCA_ denote the time spent by the droplet in constant contact radius, stick-slip and constant contact angle mode respectively [21,24]. A graph representing the same has been reprinted in Fig 5b (from Picknett and Bexon [24]) for clarity. Our experiments also suggested that for different droplet sizes (in the range 0.5 to 3 μL), evaporation duration decreases with increasing MOE_f_ or CCR mode; taken on different surfaces as shown in Fig 5a. Here, it is important to note that GDL and PDMS show nearly similar initial equilibrium contact angles (within 10° variation) but the contact line dynamics is remarkably different. MOE_f_ for PDMS is ~0.56 whereas for GDL it is ~0.86 (3μL droplet). This variation in contact line pinning (quantified by MOE_f_) effects the total evaporation time by ~300s (20%). Similar variation is shown in Fig 5c for data obtained from Shin, Dong Hwan, et al. [25]. Henceforth, the importance of contact line dynamics and its effect on physical observation like evaporation duration is comprehensively put forward by the parameter MOE_f_.

## Conclusion

MOE plots and MOE_f_ should be helpful to decipher substrate characteristics with respect to the three phase contact line dynamics. MOE plots are universal diagrams (we can plot for all drops irrespective of the substrate kind—hydrophobic or hydrophilic) that provide graphical interpretation for mode of evaporation, CAH and aid in quantifying MOE for comparative studies. In future, empirical formulations may also be developed based on MOE_f_. As research in droplet contact line dynamics progresses, mode of evaporation can be significantly important in evolving design parameters for a droplet-based system. It is anticipated that the simplicity of the approach shall be welcomed by all researchers and to be used widely for representation as well as analytical studies.

## Supporting information

S1 Supporting Information (DOCX)
