# Supplementary material for: Universal representations of evaporation modes in sessile droplets
Source: PLoS One. 2017 Sep 15;12(9):e0184997. doi: 10.1371/journal.pone.0184997 (PMC5600401; doi:10.1371/journal.pone.0184997)
Supplement: S1 Supporting Information — (DOCX) [file pone.0184997.s001.docx]

**Supporting Information**

**Universal representations of evaporation modes in sessile droplets**

Angkur Jyoti Dipanka Shaikeea^1^, Saptarshi Basu^2*^, Abhishek Tyagi^2^, Saksham Sharma^2^, Rishabh Hans^2­^_,_ Lalit Bansal^2^

^1^Department of Engineering Science, University of Oxford, U.K.

^2^Department of Mechanical Engineering, Indian Institute of Science, Bangalore, India

* Corresponding author

Email – id: [sbasu@mecheng.iisc.ernet.in](mailto:sbasu@mecheng.iisc.ernet.in)

**Experimental Details**


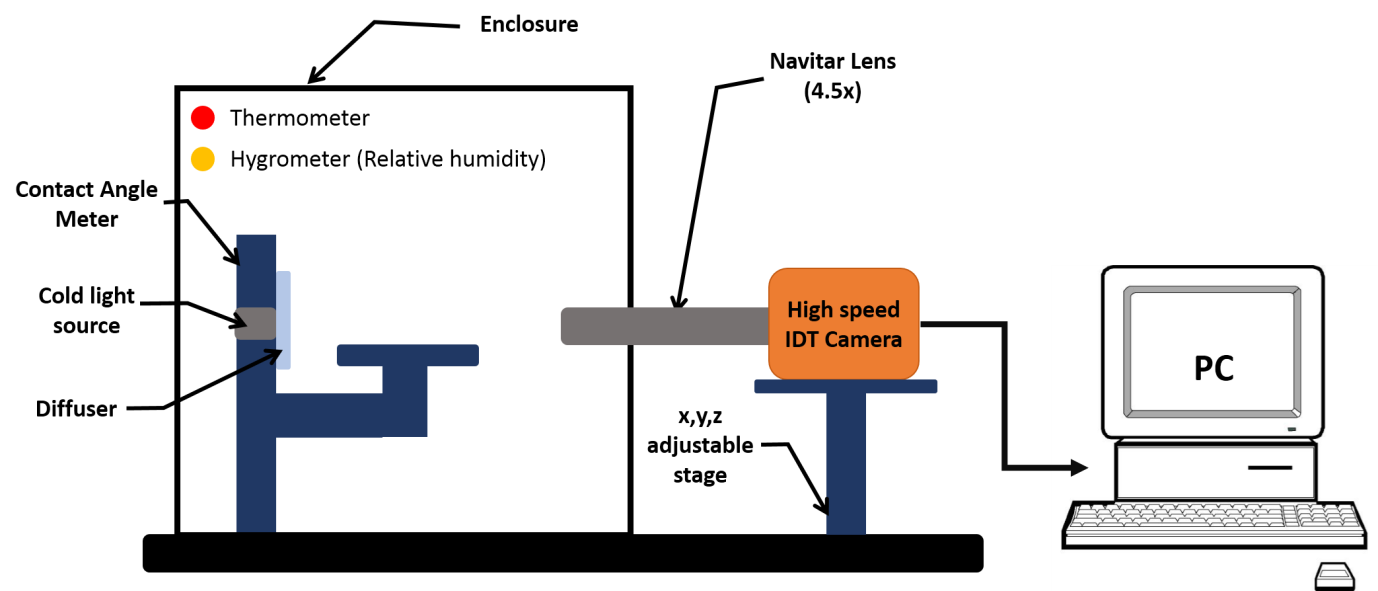


**S1 Fig.** Experiment setup.

Droplets are placed on a horizontal platform (contact angle meter) which is adjustable along the three coordinate axes of space. Shadowgraph images are obtained with cold backlit illumination passed through a diffuser. Images are acquired through a digital camera (Nikon DSLR D7200) fitted to a Navitar 4.5x zoom lens (spatial resolution 2.8 µm/pixel). Images are post processed in Image J software to measure the contact angle and contact radius. The setup is enclosed to prevent variations in relative humidity as maintained by a dehumidifier (Novita Solutions) and measured using Thor Labs hygrometer.

**Substrate Preparation**

Polydimethylsiloxane (PDMS): Sylgard 184 pre polymer and cross linker are mixed in the weight ratio 10:1. The mixture is then spin coated on glass slides and cured in oven for 4 hours at 90°C.

Gas Diffusion Layer (GDL): It is a carbon paper with 10 % PTFE coating bought from Sainergy Fuel Cell India Pvt. Ltd.

Superhydrophobic (SH) : It is prepared by coating a liquid repelling solution procured off the shelf.

**Substrate Properties**

**S1Table.** Properties of substrates.

| **Properties**  **Substrates** | **Apparent Contact**  **Angle** | **Contact Angle Hysteresis** | **Roughness**  **(microns)** |
| --- | --- | --- | --- |
| **PDMS** | 115° | 7° | 0.04 |
| **GDL** | 125° | 14° | 12 |
| **SH** | 165° | 2° | 1 |

**Area calculation for stick-slide (ar (SS)) from MOE plot**

**
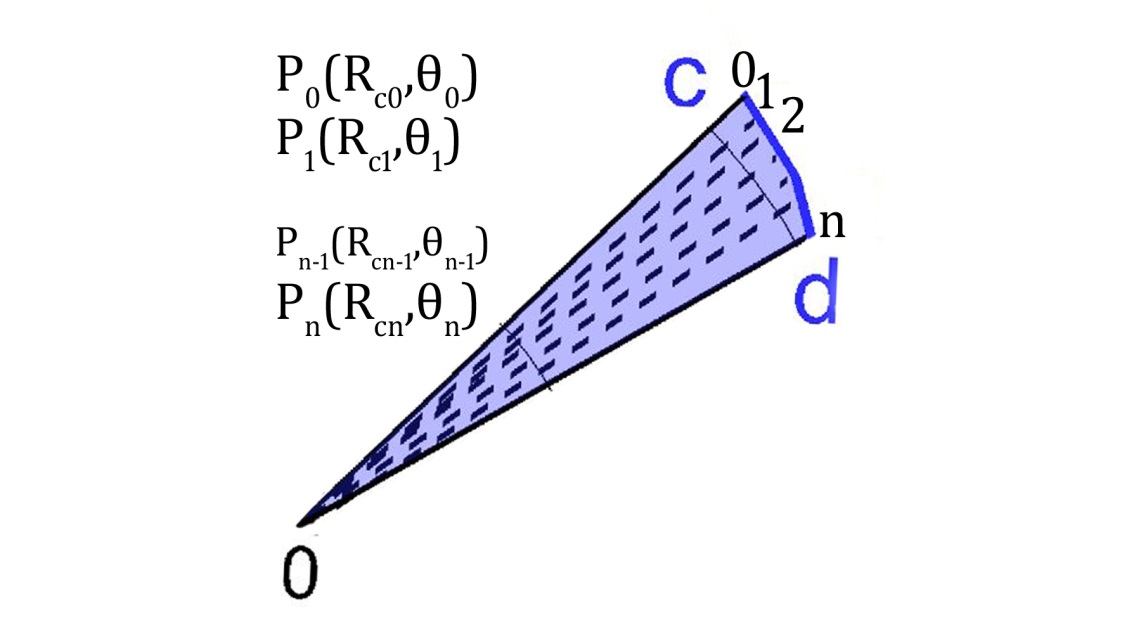
**

**S2 Fig.** Meshing of the sector c0d (undergoing SS) into circular sectors.

The locus P(R(t),θ(t)) during SS mode is non-circular and hence formula for area of a circular sector cannot be directly applied. Here, non-circular $\hat{cd}$ is segmented with the help of data points to form approximately circular sectors. Suppose, there are n+1 data points of P, measured in the SS event (cd). Then area under $\hat{cd}$ is obtained as tabulated below.

**S2 Table.** Area calculation table.

|  | **R_c_** | **θ_c ­_(in rad)** | **R_i_** | **Δθ_i_** | **Δarea_i_=[(Δθ_i_)/2]R_i_^2^** |
| --- | --- | --- | --- | --- | --- |
| **P_0_** | R_c0_ | θ_c0_ |  |  |  |
| **P_1_** | R_c1_ | θ_c1_ | $=\frac{(R_{c0}^{2}+R_{c1}^{2})}{2}$ | = θ_c0_ - θ_c1_ | $=\frac{{\Delta\theta}_{1}}{2} R_{1}^{2}$ |
| **P_2_** | R_c2_ | θ_c2_ | $=\frac{(R_{c1}^{2}+R_{c2}^{2})}{2}$ | = θ_c1_ - θ_c2_ | $=\frac{{\Delta\theta}_{2}}{2} R_{2}^{2}$ |
| **..** | .. | .. | .. | .. | .. |
| **P_n-1_** | R_c n-1_ | θ_c n-1_ | $=\frac{(R_{c n-1}^{2}+R_{c n-1}^{2})}{2}$ | = θ_c n-1_ - θ_c n-2_ | $=\frac{{\Delta\theta}_{n-1}}{2} R_{n-1}^{2}$ |
| **P_n_** | R_c n_ | θ_c n_ | $=\frac{(R_{c n}^{2}+R_{c n}^{2})}{2}$ | = θ_c n_ - θ_c n-1_ | $=\frac{{\Delta\theta}_{n}}{2} R_{n}^{2}$ |
| **Area under** $\hat{\boldsymbol{cd}}$ | | | | | $\mathbf{=}\sum_{\mathbf{i=1}}^{\mathbf{n}} \mathbf{area}_{\mathbf{i}}$ |

It is to be noted here that as the number of data points increases, the approximated circular sectors become smaller and hence circular assumption is more accurate. Matlab/Python codes can be written to perform the same from a datasheet.
